# Proteome data to explore the axolotl limb regeneration capacity at neotenic and metamorphic stages

**DOI:** 10.1016/j.dib.2020.105179

**Published:** 2020-01-28

**Authors:** Turan Demircan, Mustafa Sibai, Ebru Altuntaş

**Affiliations:** a Muğla Sıtkı Koçman University, School of Medicine, Department of Medical Biology, Turkey; b Regenerative and Restorative Medicine Research Center, REMER, Istanbul Medipol University, Istanbul, Turkey; c Muğla Sıtkı Koçman University, Graduate School of Natural and Applied Sciences, Turkey

**Keywords:** Axolotl, Regeneration, Proteomics, LC-MS/MS, Metamorphosis

## Abstract

The presented data article reports protein expression profiles during a time course of limb regeneration in the highly regenerative neotenic and regeneration-deficient metamorphic axolotl (*Ambystoma mexicanum*). A protein database was first generated from transcriptome data, which was used concomitantly with nanoLC-MS/MS to identify and assess significant changes of protein levels among 0, 1, 4, and 7 days post-amputation (dpa) in both animal stages, yielding a total of 714 significant differentially expressed proteins. Gene ontology categories of these identified proteins were examined in terms of biological processes, molecular function and cellular components. Innate clustering patterns of the samples were investigated using hierarchical clustering and were visualized on a heatmap. The data reported here constitutes an extension of “Comparison of protein expression profile of limb regeneration between neotenic and metamorphic axolotl” article Sibai et al., 2019 [1]. The associated mass spectrometry raw data have been deposited in the ProteomeXchange Consortium (http://proteomecentral.proteomexchange.org) with the dataset identifier PXD014806.

**Table:** Specifications Table

Subject	Proteomics
Specific subject area	Label-free proteome profiling of axolotl limb regeneration
Type of data	Figures, Table
How data were acquired	NanoLC-MS/MS system (Acquity UPLC M-Class and SYNAPT G2-si HDMS; Waters)
Data format	Raw and analysed
Experimental factors	Neotenic and metamorphic axolotl limb
Experimental features	*Axolotls used in this study were chosen randomly among the siblings. Half of the animals were kept in neoteny and the other half is induced to metamorphosis by thyroid hormone administration. Limb amputation of both neotenic and metamorphic animals was followed by sample collection at 0, 1, 4 and 7 dpa. Proteins were extracted from these collected samples.*
Data source location	Muğla Sıtkı Koçman University, Muğla, Turkey
Data accessibility	Data related to this article were deposited to the: 1-) ProteomeXchange Repository name: PRIDE Data identification number: PXD014806 Direct URL to data: https://www.ebi.ac.uk/pride/archive/projects/PXD014806 2-) Figshare Repository name: Figshare Data identification number: 10.6084/m9.figshare.11492319 Direct URL to data: https://figshare.com/s/b5049269e4384ce8ec3b
Related research article	Sibai M., Altuntaş E., Süzek BE., Şahin B., Parlayan C., Öztürk G., Baykal AT., Demircan T., Comparison of protein expression profile of limb regeneration between neotenic and metamorphic axolotl, Biochemical and Biophysical Research Communications, 2019, https://doi.org/10.1016/j.bbrc.2019.11.118

**Table:** 

**Value of the Data** • This data describes for the first time the characterization of the differences and similarities in the protein expression profile of regeneration-permissive neotenic and regeneration-deficient metamorphic limbs. • Since the regeneration capacity of axolotl decreases with metamorphosis [2], this data could be of interest to research groups studying molecular basis of the regeneration by offering an insightful dataset to compare the proteome of regenerative and non-regenerative forms of axolotl. • The dataset provides a useful platform for further functional studies to elucidate the pathways and key regulators involved in limb regeneration. • The metamorphic axolotl dataset is a valuable resource for understanding the gene expression alteration after metamorphosis.

## 1 Data

**Fig. 1:**
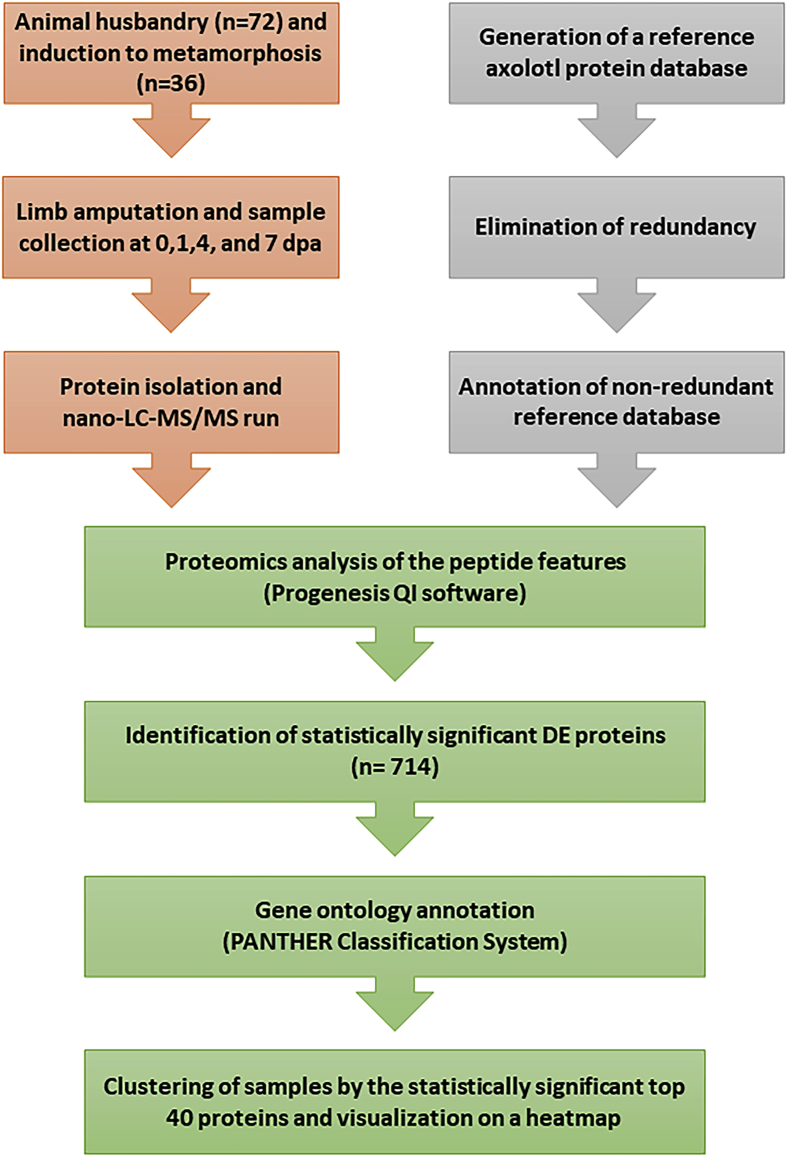
Workflow describing experimental design. Samples from amputated tissues were collected from neotenic and metamorphic axolotls at 0,1,4, and 7 dpa. A reference protein database was then generated to be used in proteomic analyses of the collected samples and the subsequent downstream analyses of differential expression, gene ontology and clustering analyses.

**Fig. 2:**
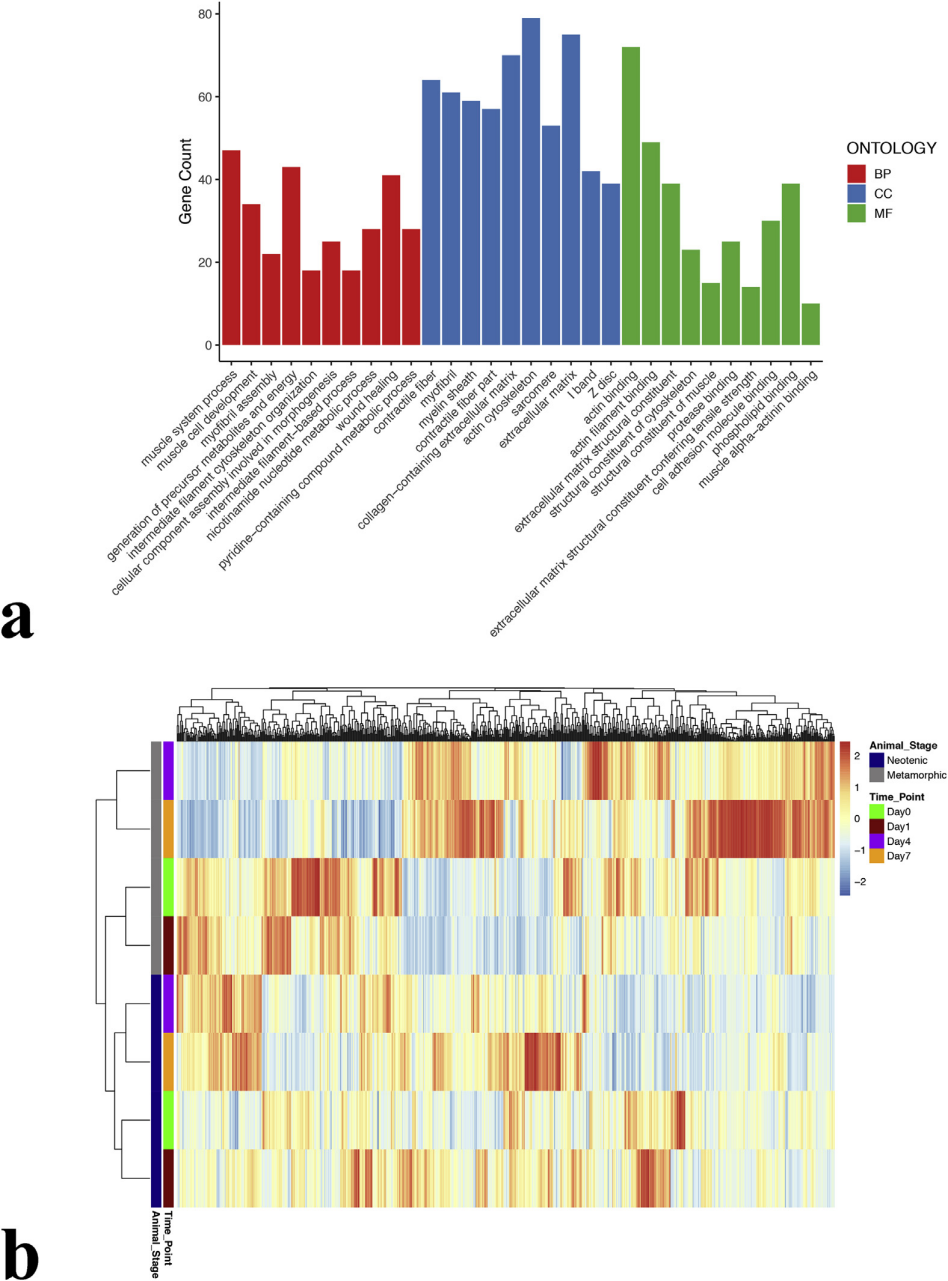
Gene ontology and sample clustering. a) The list of 714 DE proteins were tested for their putative roles in different gene ontologies (biological processes, molecular functions, cellular components) using the R environment. b) The list of 714 DE proteins were visualized in a 2D-heatmap clustering the proteins and samples by their animal stage and time point annotations based on agglomerative hierarchical clustering algorithm.

To investigate the differential alterations of the proteome between neotenic and metamorphic axolotl limbs during regeneration, we conducted LC-MS/MS proteomic analyses on samples obtained from limb tissues at 0, 1, 4, and 7 dpa amounting to a total of 72 samples equally collected from both animal stages (Fig. 1). In order to successfully carry out protein identification, a duplicate-free protein database was produced based on available axolotl transcriptome data (Fig. 1). The generated protein database was subsequently used as a reference to detect statistically significant proteins among the 4 amputation timepoints for both animal stages, yielding a total of 714 non-redundant proteins (p value ≤ 0.01, fold change ≥ 2.0) (Fig. 1). We then sought to holistically unravel the gene ontologies enriched by those proteins via taking the mouse protein orthologs which were tested for functional classification in R environment [3] (Fig. 2a). The whole list of gene ontologies enriched by 714 proteins were presented in Supplementary table 1 and top 10 of each list was visualized on Fig. 2a. Processes such as ‘muscle system process’, ‘generation of precursor metabolites and energy’, and ‘wound healing’ terms were highly represented in the biological processes enriched by those proteins. As for the molecular functions, most of the proteins were related to ‘actin binding’ and ‘actin filament binding’ and ‘phospholipid binding’ functions. Cellular components ontologies such as ‘extracellular matrix’, ‘actin cytoskeleton’ and ‘collagen−containing extracellular matrix’ constituted the majority of the terms enriched by this dataset. PANTHER system [4] was also applied to generate a more generic overview of gene ontology classification enriched by those proteins (Supplementary figure 1a). Last but not least, we were interested in uncovering putative natural clusters within the dataset that might shed light on the actual biological similarities and differences in axolotl limb regeneration between the two developmental stages, as well as timepoint-based regeneration. To achieve this purpose, we generated a 2D hierarchical clustering-based heatmap showing all 714 identified proteins (Fig. 2b), followed by the top 40 significant proteins among all conditions (Supplementary figure 1b). Both heatmaps showed consistent patterns. According to the agglomerative clustering approach, the first two timepoints (0, 1 dpa) tend to cluster together, as do the last two timepoints (4, 7 dpa) in both animal stages. Going up the dendrogram, metamorphic 0 and 1 dpa samples tend to cluster together with that which combines the first two and the last two timepoint neotenic samples. The main two clusters tend to separate metamorphic 4 and 7 dpa samples from the rest.

## 2 Materials and methods

### 2.1 Experimental design, induction to metamorphosis and sample collection

Growth and breeding of axolotls obtained from the Ambystoma Genetic Stock Center were conducted at Istanbul Medipol University Medical Research Center. Housing of the animals was carried out in %40 Holfreter's solution. The feeding protocol composed of Staple food (JBL Novo LotlM, Neuhofen, Germany), feeding animals once per day. The animals were contained each per aquarium on a 12:12 light-dark cycle at a constant temperature (18–20 °C). Every protocol and experimental procedure pertaining to animal usage was approved by the local ethics committee at Istanbul Medipol University (authorization number 38828770-E15936). Adult wildtype axolotls (n = 72, 12–15 cm in length, 1 year old) were randomly chosen from siblings for this experiment. Axolotls were divided into two groups, half of which were kept in neoteny and the other half were induced to metamorphosis using L-thyroxine (Sigma-Aldrich, St Louis, MO, USA, Cat. No. T2376) as described in Ref. [5]. L-thyroxine was dissolved in Holtfreter's solution equivalent to 50 nM final concentration, though which T4 solution was prepared. Every third day, freshly prepared T4-containing solution was used to replace the animals' rearing solution. Having been treated with T4 solution for 6 weeks, animals start to exhibit features such as weight loss as well as disappearance of fin and gills. We had Metamorphic axolotls adapt to terrestrial life conditions for a month in the absence of any hormone treatment, which was followed by limb amputations and sample collection.

Subgroups based on different amputation timepoints (0, 1, 4, and 7 dpa) were randomly formed from the 36 neotenic animals. In order to inspect repeatable accuracy, three biological replicates (R1, R2, and R3) were generated from 9 animals per group. In each biological replicate, samples of 3 animals were pooled together to eliminate inter-individual variations. Metamorphic axolotls were similarly grouped. 0.1% Ethyl 3-aminobenzoate methanesulfonate (MS-222, Sigma-Aldrich, St Louis, MO, USA) was used as an anesthetic prior to amputations. Amputations were performed on the right forelimb of each animal at mid-zeugopod level as described in Ref. [6]. Samples collected at 0 dpa and 1 dpa were cut from approximately 1-mm tissue around the amputation site. Samples collected at dpa4 and dpa7 were cut from the newly formed blastema (0.5 mm posterior tissue from the amputation site). Liquid nitrogen was used to cryopreserve all post-collection tissue samples, storing them at −80 °C until proteomic analyses.

### 2.2 Protein extraction and sample preparation

Previously published protocols were used for sample preparation prior to LC-MS/MS [7]. UPX protein extraction buffer (Expedeon) was used for protein extraction as per the manufacturer's instructions. The samples were subjected to mechanical homogenization using a mini disposable micropestle. The samples were incubated with 200 μl UPX buffer, sonicated in 0.5 second bursts at %50 power for 1 minute using a vial tweeter (Hielscher UP200St), and placed in a 100 °C water bath for 5 minutes. Insoluble fractions were removed post-homogenization by centrifugation at 14.000 rpm for 10 minutes. Filter aided sample preparation (FASP) method was used to obtain Tryptic digest [8]. Reduction of ∼50 μg of protein lysate was performed with dithiothreitol (DTT) and incubation of protein lysate with iodoacetamide (IAA) was followed for alkylation. Subsequently, Bradford Protein Assay was applied to determine protein concentration prior to trypsinization step. As a next step, a 1:50 (w/w) of Trypsin (Promega) was used to digest the protein lysate for 18 h. Prior to LC-MS/MS analysis, Quantitative Fluorometric Peptide Assay (Pierce) was employed to measure peptide concentrations.

### 2.3 Label-free quantitative nano-LC-MS/MS proteomics analysis

LC-MS/MS-based differential protein expression analysis was performed as described in Ref. [9]. nanoLC-MS/MS system (Acquity UPLC M-Class and SYNAPT G2-si HDMS; Waters. Milford, MA, USA) was used to analyze tryptic peptide mixture (200 ng). Equilibration of columns with 97% mobile phase A (0.1% Formic Acid (FA) in LC-MS-grade water (Merck)) was carried out in addition to setting the column temperature to 45 °C. Calibration of the mass spectrometer was applied with a MS/MS spectrum of [Glu1]-Fibrinopeptide B human (Glu-Fib) solution (100 fmol/uL) conveyed through the reference sprayer of the NanoLockSpray source.

A linear 2-h gradient (4%–40% Acetonitrile 0.1% (v/v) FA, 0,300 μl/min flow rate) was set to separate the peptide samples from the trap column (Symmetry C18 5μm, 180μm i.d. × 20 mm) onto the analytic column (CSH C18, 1.7 μm, 75 μm i.d. × 250 mm). To obtain a lock mass reference at 0,500 μl/min flow rate with 60 s intervals, 100 fmol/ul Glu-qfibrinopeptide-B was used. Full scan mode for 50–2000 m/z was used in positive ionization mode, and the required acquisition parameters were used as in Ref. [9]. Data independent acquisition mode (DIA) was implemented on the MS scan using 10 V (low collision energy) as well as the MS/MS scan using 30 V (high collision energy), with a cycle time of 1.4. Ion mobility separation (IMS) was used to seperate the ions based on their drift-time. The whole IMS cycle had a wave velocity ramp applied onto it from 1000 m/s to 550 m/s. Mobility trapping's release time was set as 500 μs, with a trap height of 15 V and mobility extract height of 0 V. For the mobility separation, IMS delay was set to 1000 μs after trap release. Fragmentation of all the ions within 50–2000 m/z range was applied without any precursor ion preselection in resolution mode.

### 2.4 LC-MS/MS data processing

To analyze the peptide features quantitatively and identify the proteins, we used Progenesis QI for proteomics (v.4.0, Waters) software. Retention time alignment to a reference sample, normalization considering all proteins, as well as peptide analysis were major steps of the data analysis. For the low energy threshold 150 counts and for the elevated energy threshold 30 counts were set as processing parameters. The principle of the search algorithm has been documented in detail previously [10]. All of the acquired mass data were imported to Progenesis QI and data analysis was implemented using the following parameters: minimum number of fragmented ion matches per peptide = 3, minimum number of fragment ion matches per protein = 7, minimum number of unique peptides per protein = 2, maximum number of one missed cleavage for tryptic digestion, fixed modification = carbamidomethyl C, variable modifications = oxidation M and deamidation N and Q, false discovery rate (FDR) ≤ 1%. Only features comprising charges of 2+ and 3+ were selected.

Since there is no axolotl protein database, previously assembled Axolotl mRNA sequencing data [11] was used to generate a protein database (Supplementary files 1 and 2) in multiple steps which is explained in detail in Ref. [1]. The generated database was used as a reference for the following data processing steps. The sample sets were normalized, based on the total ion intensity. Relative quantitation of non-conflicting peptides was used in protein normalization and the statistical package in Progenesis QI software was implemented to calculate expressional changes and p values. Significance level among groups were calculated by ANOVA method and resulting dataset with p-value ≤ 0.01 were considered statistically significant. This list was further filtered and differentially expressed proteins (fold change > 2.0 between two conditions) were used in downstream analyses. The list of the identified proteins with their respective identifiers and corresponding p values can be found in Supplementary table 2. Due to the existence of a certain number of redundant identifiers, another list (which was used for downstream analyses) was created with the corresponding mouse orthologues and their gene symbols (Supplementary table 3). Gene symbols for corresponding orthologues were generated using the ClusterProfiler R package [3].

### 2.5 Gene ontology (GO) analyses

To obtain a generic overview of our dataset, the found set of proteins were used to identify the gene ontology annotation spanning the three categories; biological processes, molecular functions, and cellular components. For this purpose, first, a list of mouse orthologous of all annotated proteins were generated and, subsequently, the PANTHER Classification System [4] was implemented by uploading the list to the PANTHER system.

### 2.6 Heatmap generation and clustering

To get better insights, all proteins and the most significant (based on p value) top 40 proteins in metamorphic and neotenic samples for 0, 1, 4, and 7 dpa were visualized using “pheatmap” package in R (version: 3.6) [12,13]. The distance measure that was used in clustering the samples was “Manhattan” distance. The genes and samples were hierarchically clustered using “Complete Linkage” clustering method.
