# Supplementary material for: Proteome data to explore the axolotl limb regeneration capacity at neotenic and metamorphic stages
Source: Data Brief. 2020 Jan 28;29:105179. doi: 10.1016/j.dib.2020.105179 (PMC7005509; doi:10.1016/j.dib.2020.105179)
Supplement: Multimedia component 2 [file mmc2.docx]

**Proteome data to explore the axolotl limb regeneration capacity at neotenic and metamorphic stages**

Turan Demircan^1,2 *^, Mustafa Sibai^3^, Ebru Altuntaş^3^

1 Muğla Sıtkı Koçman University, School of Medicine, Department of Medical Biology

2 Regenerative and Restorative Medicine Research Center, REMER, Istanbul Medipol University, Istanbul, Turkey

3 Muğla Sıtkı Koçman University, Graduate School of Natural and Applied Sciences

***Corresponding author(s)**

Turan Demircan: turandemircan@gmail.com


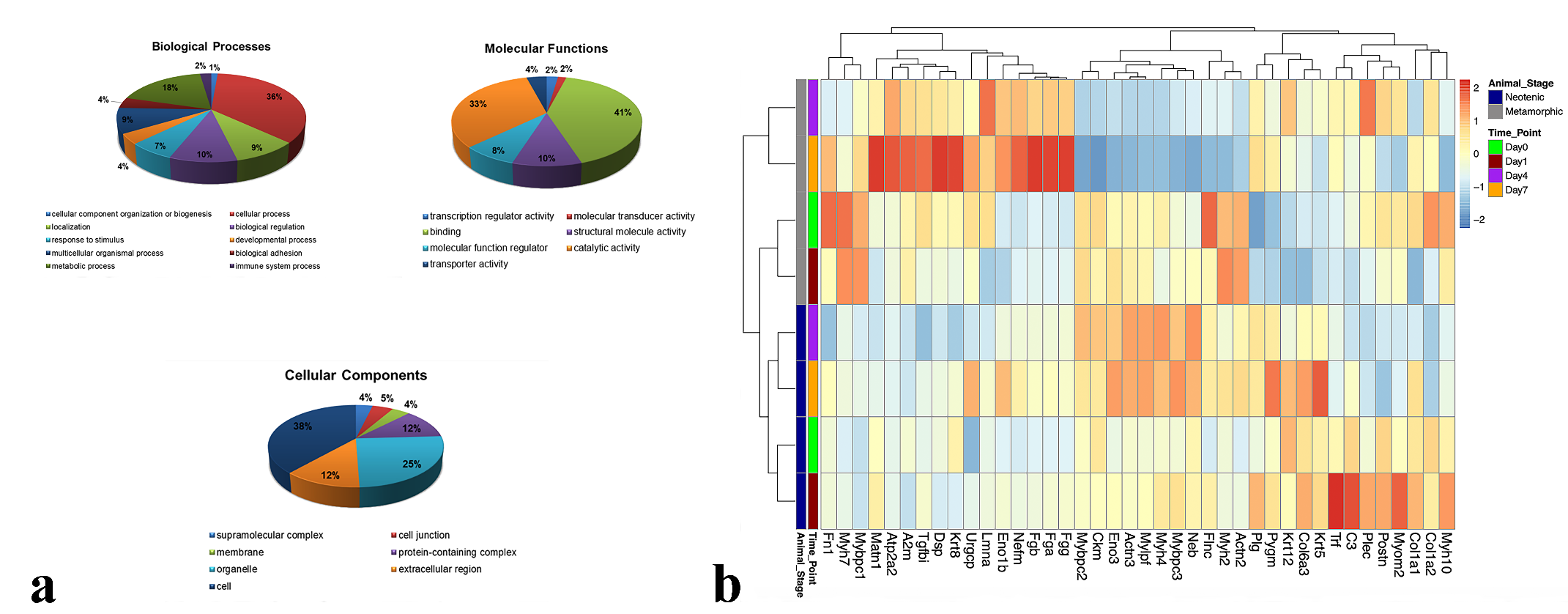


**Supplementary Figure 1:**PANTHER classification and top 40 DE proteins-based sample clustering. **a)**A more generic gene ontology classification of 714 DE proteins was generated using the PANTHER classification system. **b)**The top statistically significant 40 DE proteins were visualized in a 2D-heatmap clustering the proteins and samples
